# WGCNA Co-Expression Network Analysis Reveals ILF3-AS1 Functions as a CeRNA to Regulate PTBP1 Expression by Sponging miR-29a in Gastric Cancer

**DOI:** 10.3389/fgene.2020.00039

**Published:** 2020-02-14

**Authors:** Zhen-Hu Ren, Gao-Pan Shang, Kun Wu, Chuan-Yu Hu, Tong Ji

**Affiliations:** ^1^ Department of Oral and Maxillofacial & Head and Neck Oncology, Shanghai Ninth People's Hospital, Shanghai Jiao Tong University School of Medicine, Shanghai, China; ^2^ Department of Neonatology, Children's Hospital of Fudan University, Shanghai, China; ^3^ Stomatology Center, Tongji Hospital, Tongji Medical College, Huazhong University of Science and Technology, Wuhan, China

**Keywords:** gastric cancer, WGCNA, co-expression network, miRNA, prognosis

## Abstract

Gastric cancer (GC) is one of the most common types of human cancers worldwide. However, the detail mechanisms underlying GC progression remained to be investigated. The present study identified 2823 differently expressed mRNAs and 441 differently expressed lncRNAs in GC. WGCNA was conducted to identify highly correlated lncRNAs and mRNAs. Bioinformatics analysis observed that these dysregulated lncRNAs were significantly associated with the regulation of angiogenesis, cell division, cell-cell adhesion, blood vessel development, adaptive immune response, gastric acid secretion, immune response. Co-expression analysis identified ILF3-AS1 was a key lncRNA involved in regulating GC progression. Loss of function assays showed that knockdown of ILF3-AS1 significantly suppressed GC cell proliferation and metastasis. Mechanically, the results indicate that ILF3-AS1 could enhance PTBP3 expression as an miR-29a sponge, thereby promoting the proliferation and metastasis of GC cells. Our work suggests that the ILF3-AS1/miR-29a/PTBP3 axis may be a potential target for the clinical diagnosis and treatment of GC.

## Introduction

Gastric cancer (GC) is one of the leading causes of cancer-related deaths worldwide (Stock and Otto, 2005; Nobili et al., 2011). Moreover, GC is one of the most common gastrointestinal cancers in China (Wang and Shou, 2014). Over the past decades, a series of regulators were revealed to be associated with the progression of GC, including HER2, TP53 and NF-κB1. For example, loss of NF-κB1 causes GC with aberrant inflammation and expression of immune checkpoint regulators in a STAT-1-Dependent Manner (O'Reilly et al., 2018). However, the prognosis for advanced stage GC patients remains poor (Im et al., 2011). The 5-year survival rate is about 20%–30% (Ajani et al., 1991). Therefore, additional research is needed to discover and develop effective biomarkers and targets for gastric cancer diagnosis and treatment.

lncRNAs are a type of ncRNAs that are more than 200 bps in length (Wilkes et al., 2017). LncRNAs were observed to be dysregulated in multiple human diseases, such as diabetes, cardiovascular diseases and cancers (Yang et al., 2017). In GC, lncRNAs are involved in regulating cancer cell proliferation, migration, apoptosis and EMT progression. Several GC tumorigenesis-associated lncRNAs have been identified in recent years. For instance, lncRNA GMAN ia up-regulated in GC tissues and promotes the translation of Ephrin A1 by competitively binding GMAN-AS (Zhuo et al., 2019). HOXD-AS1 confers cisplatin resistance in GC through epigenetically silencing PDCD4 *via* recruiting EZH2 (Ye et al., 2019). Long non-coding RNA SNHG3 promotes progression of GC *via* regulating neighboring MED18 DNA methylation (Xuan and Wang, 2019). LncRNA KRT19P3 suppresses proliferation and metastasis through COPS7A-mediated NF-κB pathway in GC (Zheng et al., 2019). LINC01939 inhibits the metastasis of GC by serving as a molecular sponge of miR-17-5p to induce EGR2 expression (Chen et al., 2019). However, the roles of the majority of lncRNAs in GC remain to be further investigated,

Weighted gene co-expression network analysis (WGCNA) is a powerful tool for the identification of highly correlated genes and has been extensively adopted to identify candidate biomarkers (Zhang and Horvath, 2005). In the present study, we identified tumor-related lncRNAs in GC using WGCNA method. Bioinformatics analysis were also conducted to reveal the potential functions of GC related lncRNAs (Wang et al., 2016). Finally, ILF3-AS1 were identified to be a potential key regulator in the progression of GC. We believe that this study will provide useful information to validate ILF3-AS1 as a novel biomarker associated with GC prognosis and progression.

## Material and Methods

### Data Acquisition and Processing

The raw gene expression levels in GC were downloaded from the cBioPortal database (http://www.cbioportal.org/). The clinical information of all GC patients are included in **Supplementary Table 2**. We identified the outlier samples (to be excluded) by hierarchical cluster analysis of the normal and diseased data sets *via* the hclust function in WGCNA (Jin et al., 2019). We identified differentially expressed mRNAs (DEGs) and lncRNAs (DElncRNAs) using the Linear Models for Microarray data (Limma) package (Ritchie et al., 2015) in R. Genes with more than a 2-fold difference in expression were regarded as differentially expressed genes (DEGs, adjusted *p.*value <0.05).

### WGCNA and the Identification of Modules

The WGCNA R package was used to assess the relative importance of lncRNAs and their module membership. We first used paired Pearson correlations to evaluate the weighted co-expression relationships among the subjects in all data sets in the adjacency matrix. Then, a topological overlap matrix (TOM) similarity function was used to convert the matrix to a TOM. The resulting TOM was based on genetic similarity of biological significance and was used to measure the co-expression relationships between genes. Each TOM was used to perform hierarchical clustering analysis *via* the flashClust function in R. We followed the steps of data processing as outlined in the Horvath Lab, UCLA protocol (https://horvath.genetics.ucla.edu/html/CoexpressionNetwork/Rpackages/WGCNA/Tutorials/). The network was visualized by Cytoscape 3.7.0 software (Shannon et al., 2003) after determining the weighted correlations.

### Cell Culture

AGS and SGC-7901 cells were purchased from the Shanghai Cell Bank of the Chinese Academy of Sciences. All cells were cultured in RPMI 1640 medium supplemented with 10% fetal bovine serum (FBS) (GIBCO-BRL, Invitrogen, Carlsbad, CA, USA), 100 U/mL penicillin, and 100 mg/mL streptomycin in humidified air at 37°C with 5% CO_2_. The cells used in experiments were within 10 passages from thawing.

### RNA Extraction and Real-Time qPCR Analysis

RNA was extracted from tissues or cultured cells with TRIzol Reagent (Life Technologies, Scotland, UK) according to the manufacturer's protocol. RNA was reverse-transcribed with Prime Script RT Master Mix (Takara, catalog no. RR036A). Real-time qPCR was performed using SYBR Select Master Mix (Applied Biosystems, catalog no. 4472908) with the ABI7300 system (Applied Biosystems, Foster City, CA, USA) according to the manufacturer's instructions. The primer sequences used were as follows: 5′-TAAACCCCACTGTCTTCC-3′ (forward) and 5′-TTCCTTGCTCTTCTTGCTC-3′ (reverse) for ILF3-AS1; 5′ACAGCTAATGGGAATGACAGCA-3′- (forward) and 5′-CTGGCTTCGAAGGTGAGGAG-3′ (reverse) for PTBP3; 5′-GGAGCGAGATCCCTCCAAAAT-3′ (forward) and 5′-GGCTGTTGTCATACTTCTCATGG-3′ (reverse) for GAPDH. The ΔΔCt method was used to determine fold changes in subsequent calculations.

### RNA Isolation of Nuclear and Cytoplasmic Fractions

The subcellular localization of ILF3-AS1 was detected using the PARIS Kit according to the manufacturer's protocol (Ambion, Life Technologies, Carlsbad, CA, USA).

### siRNA, Plasmid Construction, and Cell Transfection

siRNAs were provided by GenScript (Nanjing, China). miRNA mimics and primers were provided by RiboBio (Guangzhou, China). The siRNAs and miRNA mimics were transfected using Lipofectamine iMAX (Invitrogen, Shanghai, China), and plasmids were transfected with X-tremeGENE (Roche Applied Science) according to the manufacturer's instructions. The shRNAs target sites were as follows: for si-ILF3-AS1-1: GCCTGTTGATTCAGACGTTCC; for si-ILF3-AS1-2: GCTTTGTCCTTACAAGCGTGG.

### Cell Proliferation

Cells were harvested 24 h post-transfection, and the Cell Counting Kit-8 (CCK-8) assay was used to determine cell growth according to the manufacturer's instructions (Nanjing KeyGen Biotech., Nanjing, China).

### Migration and Invasion Assays

Migration and invasion assays were conducted in Transwell chambers (Corning, Corning, USA) coated with or without Matrigel (BD Biosciences) on the upper surface of the 8-μm pore size membrane. In brief, transfected cells were harvested, suspended in serum-free medium, and cells were plated into the upper chamber for migration or invasion analysis, respectively, and medium containing 10% FBS was placed into the lower chamber. After incubation for 24 h, the cells in migration or invasion *via* the membrane to the lower surface were fixed, stained and counted using an inverted microscope.

### Statistical Analysis

Differences between groups were assessed using a paired two-tailed student's t-test. One-way ANOVA or the nonparametric Kruskal-Wallis test was applied to assess the relationship between ILF3-AS1 expression and other characteristics. The strength of the association between continuous variables was tested with the Spearman correlation. All statistical analyses were performed using SPSS 20 software (Abbott Laboratories, Chicago, IL, USA).

## Result

### Identification of GC Progression Related mRNAs and lncRNA

**Figure 1:**
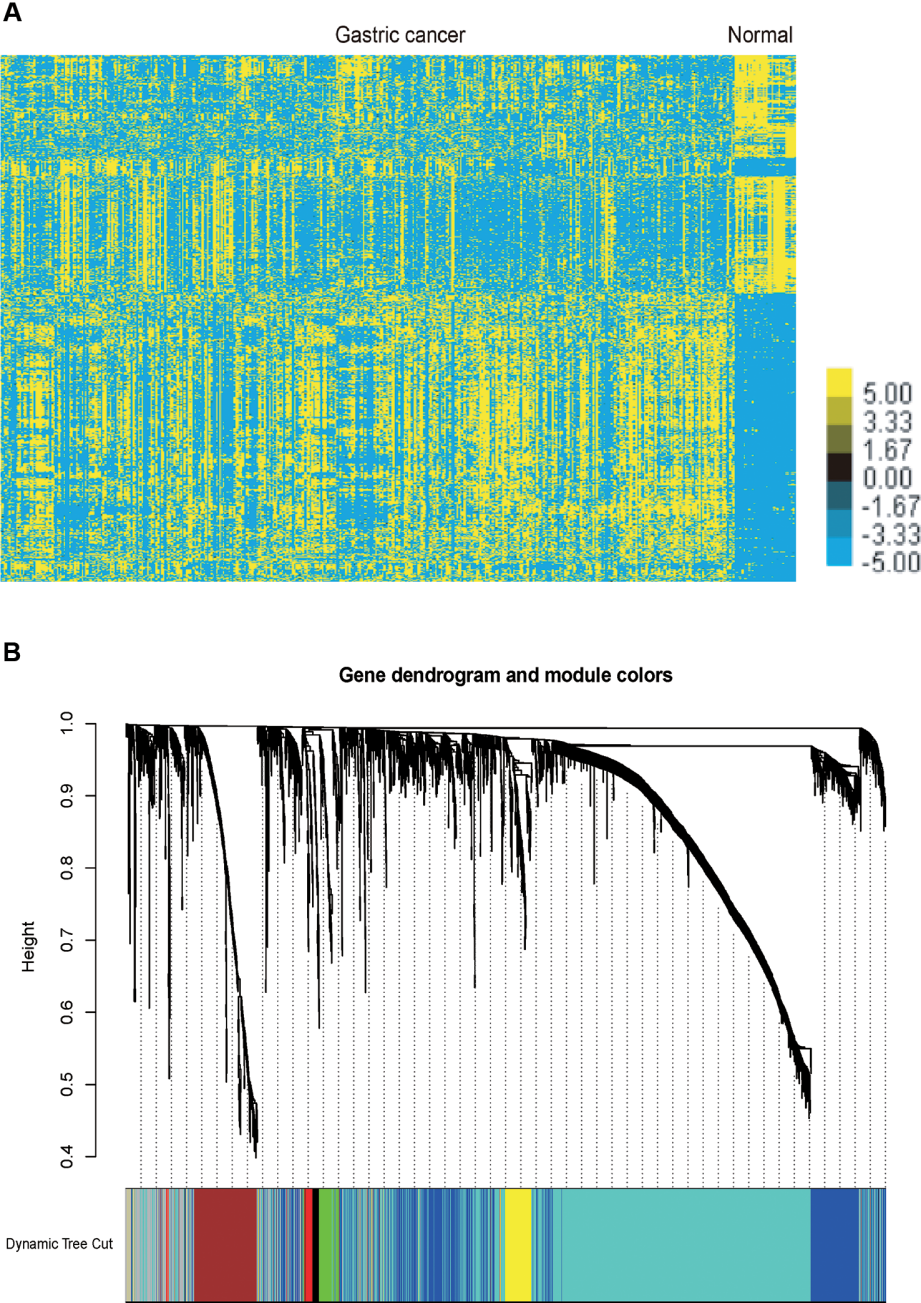
Identification of the differentially expressed mRNAs and lncRNAs in GC and gene dendrogram. **(A)** Hierarchical clustering analysis indicating the differential mRNA and lncRNA expression in GC using TCGA database. Yellow indicates high expression and Blue indicates low expression. **(B)** Gene cluster dendrogram and module colours. Gene dendrogram obtained by Topological Overlap with the corresponding module colors indicated by the color row. Each color represents the independent module which contains a group of highly correlated genes. A total of 7 modules were identified.

We performed analysis of a public dataset TCGA STAD to identify GC related mRNAs and lncRNA. TCGA STAD contained 35 normal gastric tissues and 416 GC samples. We identified 1358 up-regulated mRNAs and 1465 down-regulated mRNAs in GC samples compared to normal tissues samples. Meanwhile, we identified 254 up-regulated lncRNAs and 187 down-regulated lncRNAs in GC samples compared to normal tissues samples (**Figure 1A**).

### Identification Key Gene Modules by WGCNA

The co-expression network was constructed using the WGCNA package in R software. The results of the parameter analysis are shown in **Supplementary Figure 1**. After determining the optimal parameter (β = 4), the WGCNA algorithm was used to convert the correlation coefficient of a gene pair into the adjacent coefficient. Then, the dissimilarity of the topological overlap matrix was calculated based on the adjacent coefficient. Using the calculated dissimilarity, we carried out hierarchical analysis by agglomerative hierarchical clustering, also known as the bottom-up method. Other assumptions that we made were: (i) distances between different classes were measured by the average connectivity; and (ii) there should be at least 30 genes in each gene module. (We had tried to put this threshold smaller (< 30), but we found these small modules were of no biological significance). Based on these assumptions, we finally obtained 7 gene modules (**Figure 1B**).

### Function Annotation of GC Related lncRNAs

**Figure 2:**
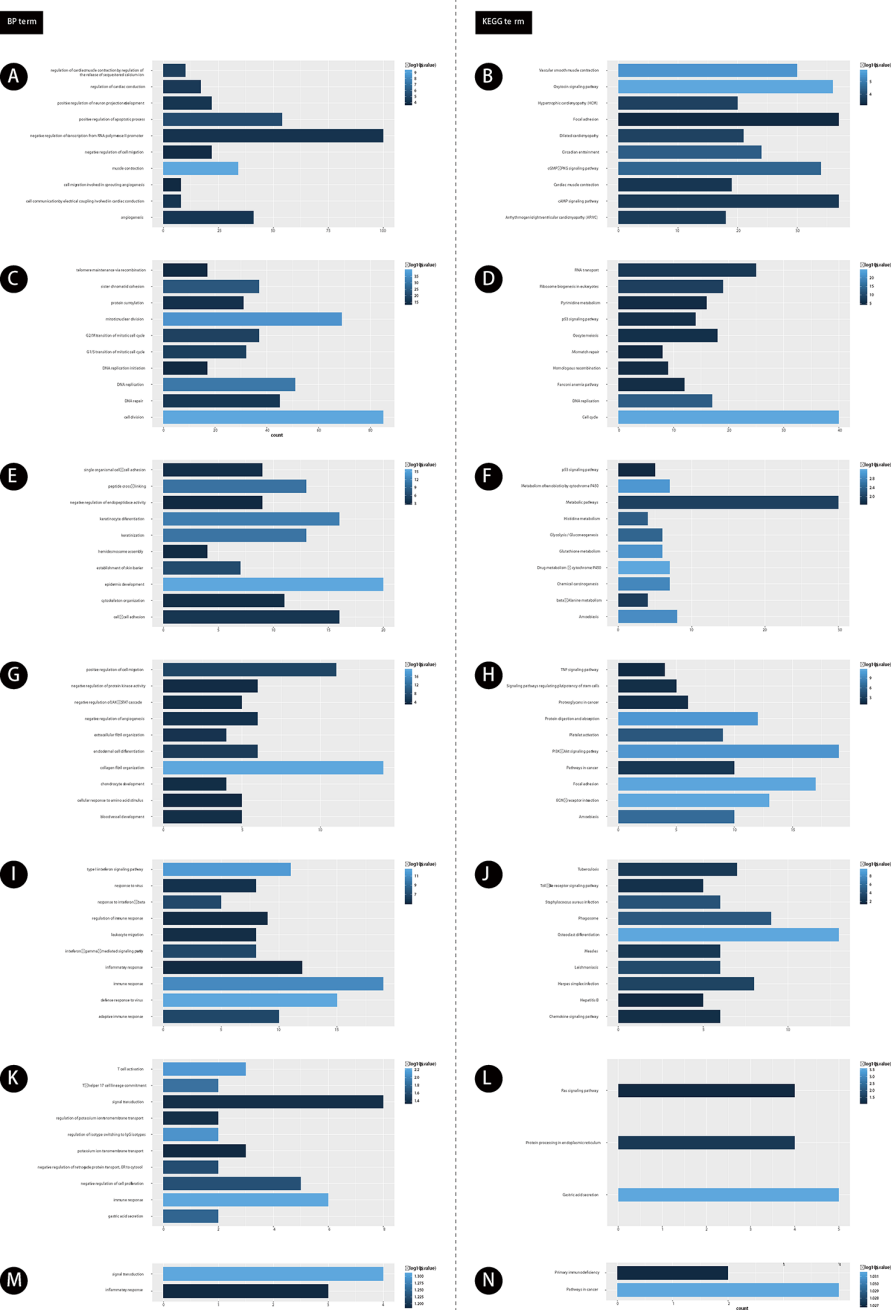
The functional enrichment analysis of genes in module 1-7. **(A**, **C**, **E**, **G**, **I**, **K**, **M)** GO analysis of gene in modules 1-7. **(B**, **D**, **F**, **H**, **J**, **L**, **N)** KEGG analysis of gene in modules 1-7. The color depth of histogram represents *p*-value. The x-axis represents gene counts. The y-axis represents the terms of GO and KEGG.

Furthermore, we performed bioinformatics analysis for GC related lncRNAs and mRNAs using DAVID system (as shown in **Figure 2**). Our results showed lncRNAs in module 1 were involved in regulating angiogenesis (**Figure 2A**). Module 2 were involved in regulating cell proliferation (**Figure 2C**). Module 3 were involved in regulating cell-cell adhesion (**Figure 2E**). Module 4 were involved in regulating blood vessel development (**Figure 2G**). Module 5 were involved in regulating adaptive immune response (**Figure 2I**). Module 6 were involved in regulating gastric acid secretion, immune response (**Figure 2K**). Module 7 were involved in regulating signal transduction and inflammatory response (**Figure 2M**).

Furthermore, we performed KEGG pathway analysis for GC related lncRNAs using DAVID system. Our results showed lncRNAs in module 1 were involved in regulating cAMP signaling pathway (**Figure 2B**). Module 2 were involved in regulating DNA replication (**Figure 2D**). Module 3 were involved in regulating Amoebiasis and p53 signaling pathway (**Figure 2F**). Module 4 were involved in regulating TNF signaling pathway (**Figure 2H**). Module 5 were involved in regulating Herpes simplex infection (**Figure 2J**). Module 6 were involved in regulating Ras signaling pathway (**Figure 2L**). Module 7 were involved in regulating Primary immunodeficiency and Pathways in cancer (**Figure 2N**).

### Construction of GC Related lncRNA-mRNA Co-Expression Networks

**Figure 3:**
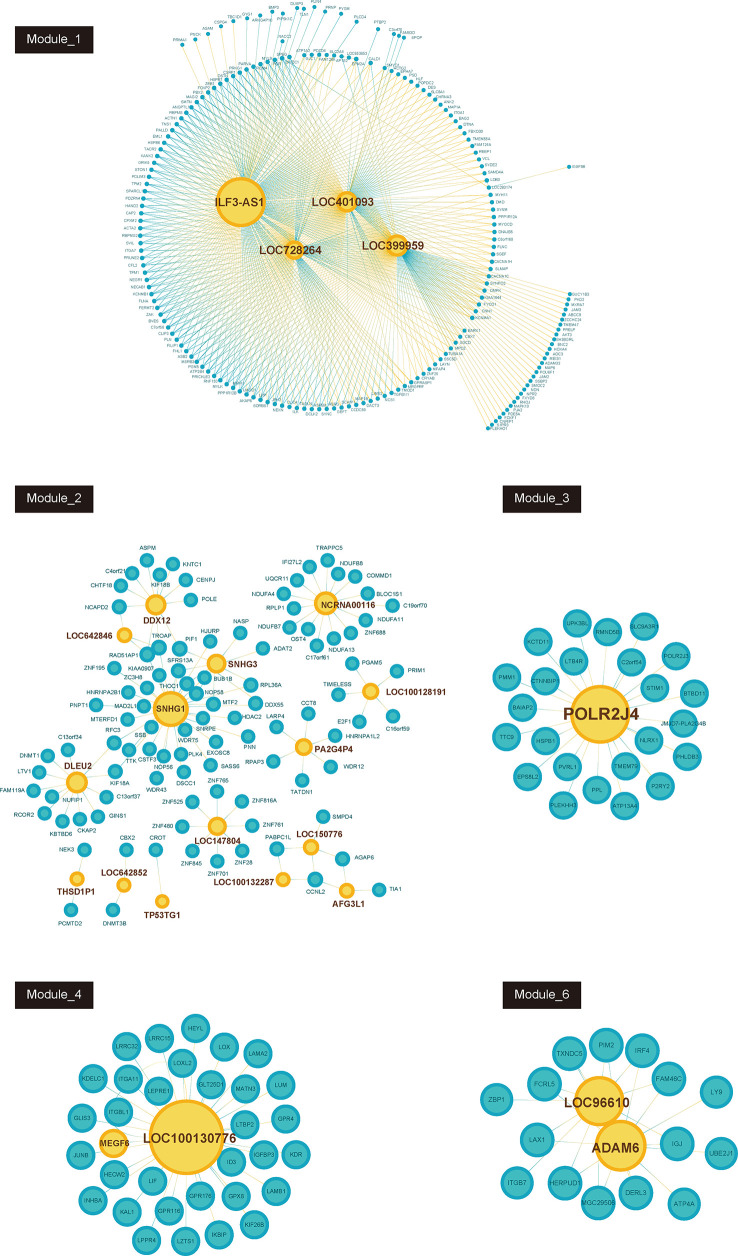
Co-expression network between lncRNAs and mRNAs in each modules (Module_1, Module_2, Module_3, Module_4, Module_6). Blue node represents lncRNA; pink node represents mRNAs. The size of node depends on the connectivity degree of node.

Furthermore, we constructed GC related lncRNA-mRNA co-expression networks by calculating the Pearson correlation coefficient of lncRNA-mRNA pairs in 7 gene modules based on WCGNA analysis. lncRNA-mRNA pairs with |R| > 0.65 were selected for co-expression networks construction (**Figure 3**). A few lncRNAs, such as ILF3-AS1, ZFAS1, SNHG1, POLR2J4, and LOC96610, were identified as key regulators in networks.

### High ILF3-AS1 Expression Correlates with Poor Survival of GC

**Figure 4:**
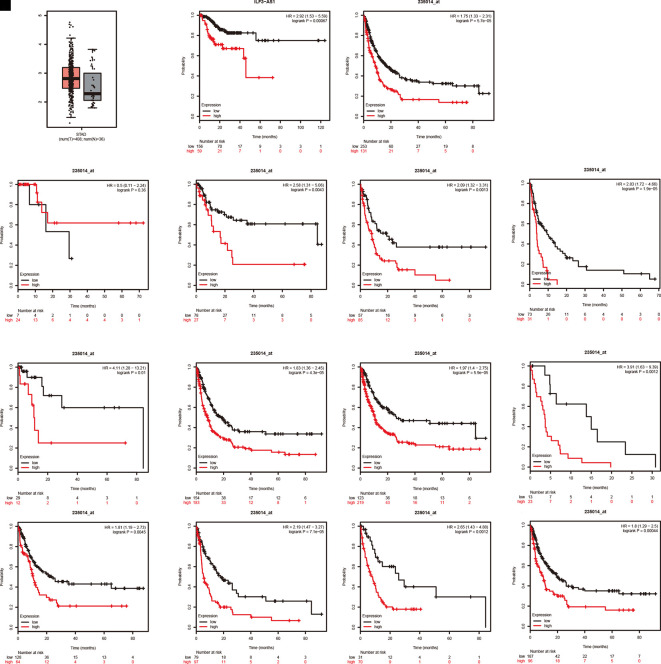
ILF3-AS1 Expression in GC and Correlates with disease free survival of GC. **(A)** The boxplot of ILF3-AS1 expression in GC between 408 tumor samples and 36 normal samples. **(B, C)** Higher expression levels of ILF3-AS1 correlates with disease free survival of GC using TCGA **(B)** and Kaplan–Meier plotter database **(C)**. **(D–O)** ILF3-AS1 expression was correlated to the disease free survival of patients with Stage 1 **(D)**, Stage 2 **(E)**, Stage 3 **(F)**, Stage 4 **(G)**, N0 **(H)**, N1+2+3 **(I)**, M0 **(J)**, M1 **(K)**, intestinal **(L)**, diffuse **(M)**, HER2 positive **(N)** and HER2 negative **(O)** GC.

The TCGA analysis showed that ILF3-AS1 was remarkably upregulated in GC tissues (**Figure 4A**). Furthermore, we validated the overexpression of ILF3-AS1 in GC using the GEPIA database. In addition, Kaplan–Meier and log-rank tests were used to analyze the relationship between ILF3-AS1 expression with disease free survival (DFS) of GC patients using TCGA and Kaplan–Meier plotter database. The results showed that a high expression of ILF3-AS1 was significantly negatively correlated with the DFS (**Figure 4B–C**) in GC patients. The GC patients with higher ILF3-AS1 expression had shorter DFS time.

Moreover, Kaplan–Meier plotter was applied to evaluate the correlation between ILF3-AS1 Expression and prognosis of patients with different types of GC cancer. Very interestingly, we found that higher expression levels of ILF3-AS1 were significantly correlated to shorter DFS time in patients with Stage 2, Stage 3, Stage 4, N0, N1+2+3, M0, M1, intestinal, diffuse, HER2 positive, and HER2 negative GC (**Figure 4E–O**). However, the dysregulation of ILF3-AS1 was not significantly correlated to the prognosis of patients with GC (**Figure 4D**). These results suggested that ILF3-AS1 could be a potential biomarker for the prognosis of GC.

### ILF3-AS1 Promotes Proliferation and Migration of GC Cells

**Figure 5:**
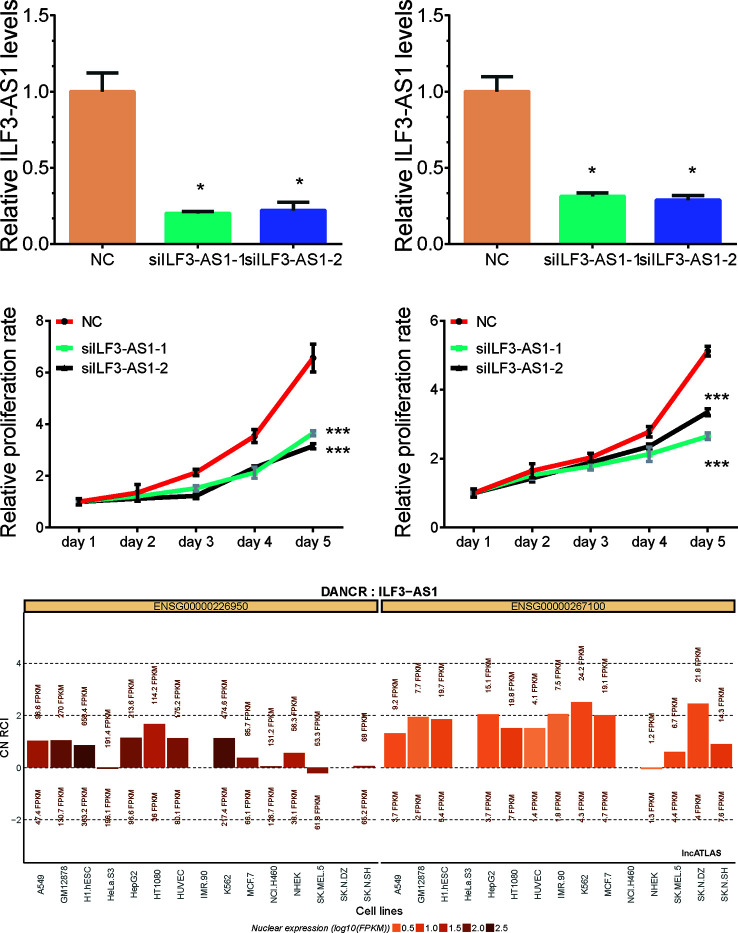
Knockdown of ILF3-AS1 induced cell proliferation in GC. **(A, B)** qRT‐PCR assay showed Knockdown results of ILF3-AS1 in SGC-7901 and AGS cells. **(C, D)** Knockdown of ILF3-AS1 induced cell proliferation in SGC-7901 and AGS cells. **(E)** By analyzing LncATLAS dataset, we found ILF3-AS1 localized in both the cytoplasm and nuclear in most of the cell lines. Data are presented as the mean ± SD (n = 3). Signifcance was defined as p < 0.05 (*p < 0.05; **p < 0.01; ***p < 0.001).

Bioinformatics analysis suggested that ILF3-AS1 might mainly impact cell cycle and proliferation. Therefore, we conducted loss-of-function assays to detect the effect of ILF3-AS1 on GC cell proliferation and migration by using Small interfering RNA (siRNA) to knock down ILF3-AS1 in GC. Two interference sequences against ILF3-AS1were transfected into SGC-7901 and AGS cells, respectively, and the expression of ILF3-AS1 was detected (**Figures 5A**, **B**). By analyzing LncATLAS dataset, we found ILF3-AS1 localized in the cytoplasm in most of the cell lines (**Figure 5E**) After knockdown of ILF3-AS1, CCK-8 assay was conducted to access cell proliferation. The data showed that proliferative rate of GC cells remarkably decreased after ILF3-AS1 knockdown (**Figures 5C**, **D**).

**Figure 6:**
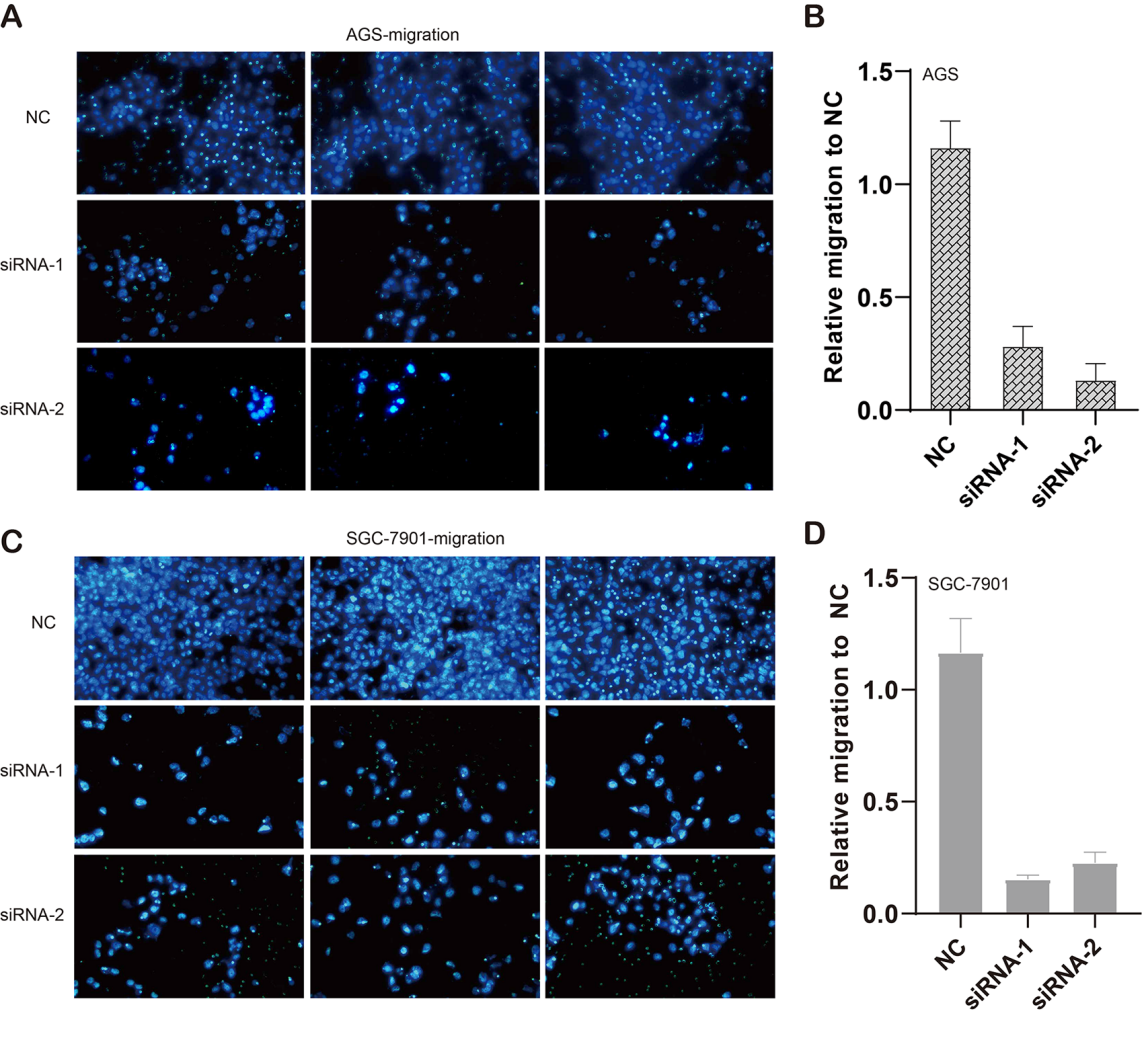
Knockdown of ILF3-AS1 induced cell migration in GC. **(A, B)** Knockdown of ILF3-AS1 induced cell migration in AGS cells. **(C, D)** Knockdown of ILF3-AS1 induced cell migration in SGC-7901 cells.

**Figure 7:**
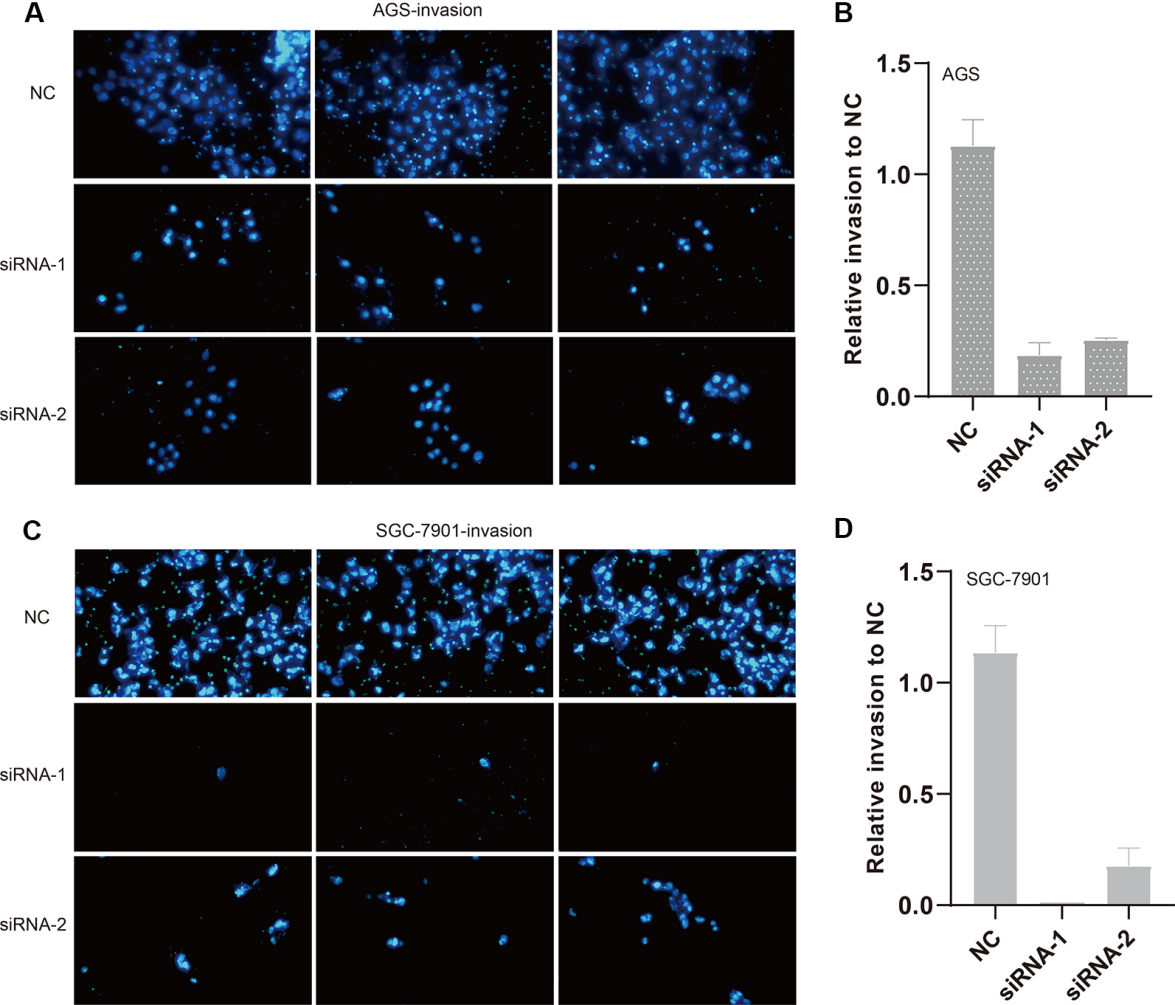
Knockdown of ILF3-AS1 induced cell invasion in GC. **(A, B)** Knockdown of ILF3-AS1 induced cell invasion in AGS cells. **(C, D)** Knockdown of ILF3-AS1 induced cell invasion in SGC-7901 cells.

We also evaluated the migratory ability mediated by ILF3-AS1 through a transwell assay. The results showed that knockdown of ILF3-AS1 suppressed the cell migration in AGS (**Figures 6A**, **B**) and SGC-7901 cells (**Figures 6C**, **D**). Next, we performed a Matrigel Transwell assay to detect the cell invasion induced by ILF3-AS1. Similarly, silence of ILF3-AS1 suppressed cell invasion in AGS (**Figures 7A**, **B**) and SGC-7901 (**Figures 7C**, **D**) cells. Taken together, ILF3-AS1 promotes cell migration and invasion in GC cells.

### ILF3-AS1 Binds with mIR-29a

**Figure 8:**
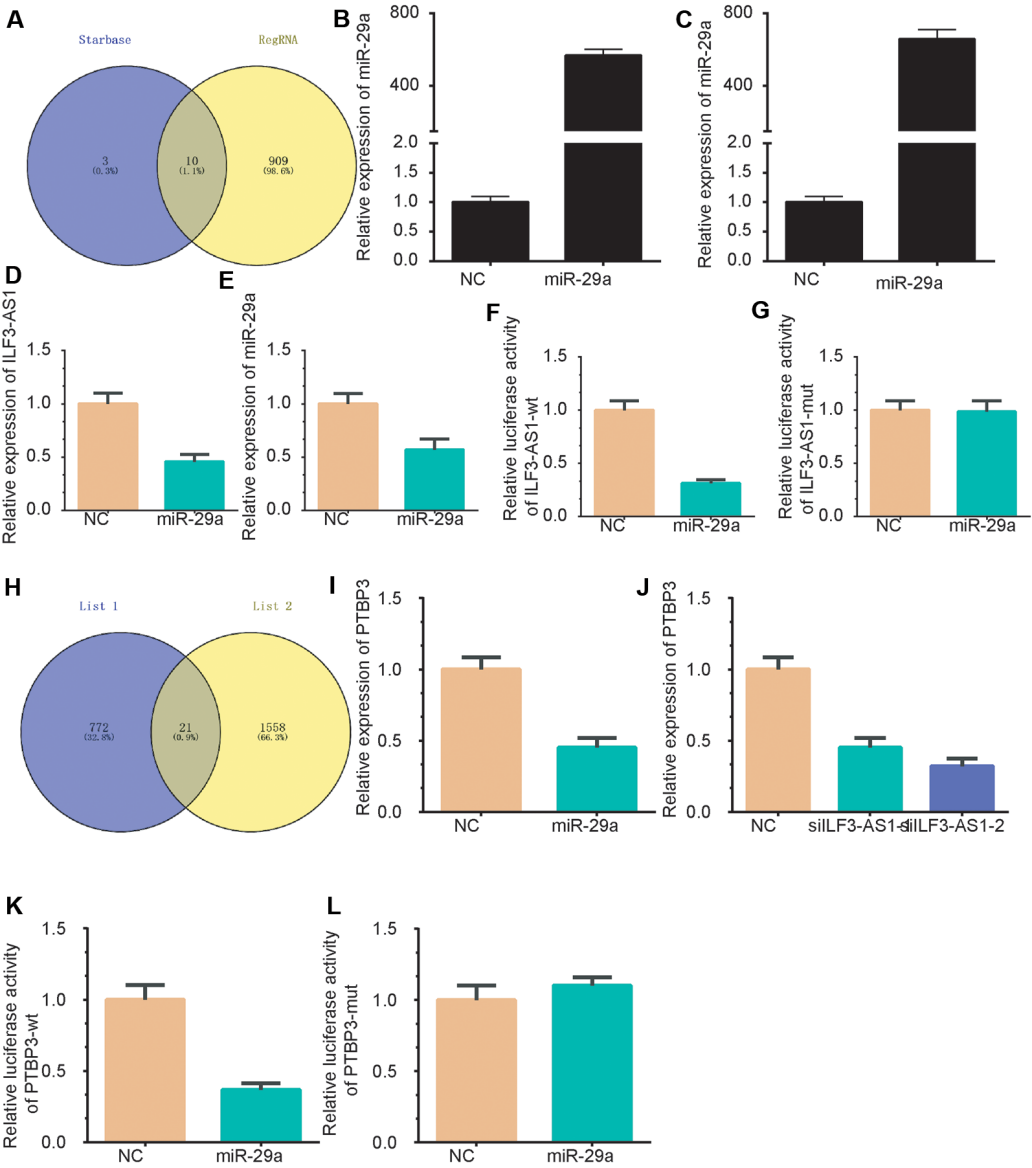
ILF3-AS1 Binds with miR-29a to promote PTBP3 expression. **(A)** A Venn plot was performed to identify the potential targeting miRNAs of ILF3-AS1 using RegRNA database and Starbase database. **(B-C)** miR-29a levels were up-regulated in AGS **(B)** and SGC-7901 **(C)** cells after transfecting with miR-29a mimics. **(D, E)** miR-29a overexpression down-regulated ILF3-AS1 expression in AGS **(D)** and SGC-7901 **(E)** cells. **(F, G)** Luciferase reporter assay showed that miR-29a significantly inhibited the luciferase activity of ILF3-AS1-wt reporter **(F)**, not ILF3-AS1-mutated reporter **(G)**. **(H)** A Venn plot was performed to identify the potential targets of ILF3-AS1 and miR-29a. **(I, J)** overexpression of miR-29a **(I)** or knockdown of ILF3-AS1 **(J)** significantly down-regulated the expression levels of PTBP3. **(K, L)** Furthermore, a dual-luciferase reporter gene assay confirmed that miR-29a could significantly inhibit luciferase activity of PTBP3-3'UTR mutated reporter **(K)**, whereas miR-29a could not significantly inhibit luciferase activity of PTBP3-3'UTR mutated reporter **(L)**.

Subcellular distribution suggests that ILF3-AS1 might have a distinct regulatory mechanism in cytoplasm. It has been proposed that lncRNA could act as competing endogenous RNA (ceRNA) in human cancer cells. Thus, we hypothesized that ILF3-AS1 may function in the ceRNA mechanism. The RegRNA database predicted that there were various miRNA binding sites within the ILF3-AS1 transcript (**Supplementary Table 1**). Together with the photoactivatable ribonucleoside-enhanced crosslinking and immunoprecipitation (PAR-CLIP) sequencing data (Chen et al., 2019), we identified that miR-29a could potentially bind with ILF3-AS1 (**Figure 8A**). The transfection efficiency was shown in **Figures 8B, C**. After miR-29a mimic transfection, the gene level of ILF3-AS1 was down-regulated compared to NC group (**Figures 8D, E**). A Luciferase reporter assay showed that miR-29a significantly inhibited the luciferase activity of ILF3-AS1-wt reporter (**Figure 8F**), not ILF3-AS1-mutated reporter (**Figure 8G**).

### PTBP3 Is a Downstream Target of mIR-29a

Based on co-expression analysis, a total of 793 predominant genes were identified to be potential targets of ILF3-AS1 (**Supplementary Table 1**). A Venn plot was performed to identify ceRNA targets of ILF3-AS1 using the target genes of miR-29a and the 568 predominant genes. As shown, 21 potential targets were identified as the potential ceRNA targets of ILF3-AS1, including SCNM1, ADAM10, PRR3, TOMM7, PURA, PTBP2, ZDHHC5, PAFAH1B2, WDFY1, TCEAL4, NUP98, CSNK1E, POLR3A, UBE2Z, ANKRD49, CLK2, MLLT11, PHLDB3, TFRC, LYSMD1, and PTBP3 (**Figure 8H**). Among these genes, PTBP3 was reported to be a key cancer metastasis regulator in GC and selected for further validation.

Very interestingly, we found that knockdown of ILF3-AS1 or overexpression of miR-29a significantly down-regulated the expression levels of PTBP3 (**Figures 8I–J**). Furthermore, a dual-luciferase reporter gene assay confirmed that miR-29a could bind to the 3′UTR of PTBP3 and significantly inhibit luciferase activity (**Figure 8K**), whereas miR-29a could not significantly inhibit luciferase activity of PTBP3-3'UTR mutated reporter (**Figure 8L**). Together, these results revealed that PTBP3 is ceRNA target of miR-29a and ILF3-AS1.

## Discussion

GC had been one of the most common types of human cancers worldwide. However, the detail mechanisms underlying GC progression remained to be investigated. The present study identified 2823 differently expressed mRNAs and 441 differently expressed lncRNAs in GC. WGCNA was conducted to identify highly correlated lncRNAs and mRNAs. Co-expression analysis identified that ILF3-AS1 was a key lncRNA involved in regulating GC progression. Loss-of-functions assays showed that ILF3-AS1 played a oncogenic role in GC by suppressing miR-29a activity to promote PTBP3 expression.

LncRNAs had been demonstrated to play a crucial role in GC. However, the roles of most lncRNAs in GC remained to be unclear. WGCNA is a systems biology approach increasingly adopted in molecular oncology. Very recently, WGCNA has been used to understand the roles of lncRNA in multiple human cancers. For example, Giulietti et al. used the WGCNA method to identify lncRNAs in pancreatic cancer and found that 11 lncRNAs were key regulators and could serve as potential prognostic biomarkers for pancreatic cancer (Giulietti et al., 2018). Zhai et al. identified recurrence−associated genes in colon cancer using the WGCNA method and found that lncRNA LINC0219 was a key lncRNA associated with the recurrence of colon cancer (Jiang et al., 2019). The present study identified 441 dysregulated lncRNAs in GC samples compared to normal tissues. WGCNA analysis revealed 6 lncRNA-mRNA co-expression modules in GC. A few lncRNAs, such as ILF3-AS1, ZFAS1, SNHG1, POLR2J4, and LOC96610, were identified as key lncRNAs in GC. Bioinformatics analysis observed that these dysregulated lncRNAs were significantly associated with the regulation of angiogenesis, cell division, cell-cell adhesion, blood vessel development, adaptive immune response, gastric acid secretion, immune response.

Long noncoding RNA ILF3-AS1 has been reported to be overexpressed in multiple human cancers. For example, ILF3-AS1 together with 14 lncRNAs were revealed to predict cervical cancer patient survival. In colon cancer, ILF3-AS1, together with LINC0184, AC105243.1, LOC101928168, MIR31HG, and AC006329.1, were revealed to be an independent predictive factor of colon cancer recurrence (Zhou et al., 2018). Bioinformatics analysis showed that ILF3-AS1 was involved in regulating proliferation and angiogenesis, and cell death in colon cancer. ILF3-AS1 was also revealed to play a crucial role in regulating cancer progression. For example, in Melanoma, lncRNA ILF3-AS1 is up-regulated in melanoma tissues and correlated with poor prognosis of melanoma patients. Functional experiments showed that knockdown of ILF3-AS1 inhibits melanoma cell proliferation, migration, and invasion through interacting with EZH2 to represses miR-200b/a/429 expression (Chen et al., 2017). In osteosarcoma, ILF3-AS1 was induced by SP1 and promoted the proliferation, migration and invasion of osteosarcoma cells through miR-212/SOX5 axis (Hu et al., 2019). However, the roles of ILF3-AS1 in GC remained to be unclear. The present study for the first time revealed that ILF3-AS1 was significantly up-regulated in GC samples compared to normal tissues. Knockdown of ILF3-AS1 significantly suppressed GC cell proliferation, migration and invasion. Mechanistically, we found that ILF3-AS1 could interacted with miR-29a to promote PTBP3 expression. Taken together, ILF3-AS1 displayed its various oncogenic roles in the progression of GC through the miR-29a/PTBP3 axis.

miR-29a is the predominant member of the miR-29 family. miR-29a was revealed to play an important regulatory role in multiple human cancers, including prostate cancer rectal cancer, and gastric cancer (Jiang et al., 2014). miR-29a acted as a tumor suppressor by regulating various biological processes including cellular proliferation, differentiation, development, and apoptosis (Li et al., 2014). In gastric cancer, MiR-29a was revealed to inhibit cell proliferation and induces cell cycle arrest through the downregulation of p42.3, suppress invasion of gastric cancer cells by targeting VEGF-A, AKT2 and Roundabout homolog 1 (Li et al., 2014). PTBP3 played an essential role in RNA splicing, 3' end processing and translation (Tan et al., 2015). PTBP3 has also been found to play important roles in lung adenocarcinoma, glioblastoma multiforme, squamous cell carcinoma, and gastric cancer. In gastric cancer, PTBP3 contributes to the cancer metastasis by mediating CAV1 alternative splicing (Liang et al., 2018). Inhibition of PTBP3 induces apoptosis and cell cycle arrest, and enhances the cytotoxicity of 5- fluorouracil in gastric cancer cells (Liang et al., 2017). The present study demonstrated, for the first time, that miR-29a/PTBP3 is regulated by ILF3-AS1 in GC. Moreover, our results show that MiR-29a is significantly down-regulated, however, PTBP3 is significantly overexpressed in GC compared to normal tissues. Higher expression of ILF3-AS1 and lower expression of MiR-29a are significantly correlated with the shorter DFS and OS in GC patients. These results suggest that MiR-29a and PTBP3, together with ILF3-AS1, could be the potential biomarkers for GC.

We should point out that there were several limitations in this study. Firstly, a rescue experiment for PTBP3 in cell proliferation and metastasis were still needed to support that ILF3-AS1 and miR-29a played their roles through PTBP3. Secondly, we only validated the molecular functions of ILF3-AS1 in GC using *in vitro* assays. We thought that the functional validation *in vivo* could further support our conclusion that ILF3-AS1 could be a potential biomarker for GC.

## Conclusion

In conclusion, we identified a total of 275 lncRNAs that were found to be dysregulated in the progression of GC. WGCNA and co-expression analysis were performed to identify highly correlated lncRNAs and mRNAs. Very interestingly, we revealed ILF3-AS1 as a key regulator in GC. The results indicate that ILF3-AS1 could enhance PTBP3 expression as a miR-29a sponge, thereby promoting the proliferation and metastasis of GC cells. Our work suggests that the ILF3-AS1/miR-29a/PTBP3 axis may be a potential target for clinical diagnosis and treatment of GC.

## Data Availability Statement

The raw gene expression levels in GC were downloaded from cBioPortal database (http://www.cbioportal.org/).

## Author Contributions

Conception and design: TJ and C-YH. Development of methodology: Z-HR. Analysis and interpretation of data: All authors. Writing, review, and/or revision of the manuscript: All authors.

## Funding

This work is supported by The Interdisciplinary Program of Shanghai Jiao Tong University (ZH2018QNA08), Shanghai Anticancer Association EYAS PROJECT (SACA-CY1B06) and Science and Technology Commission of Shanghai Municipality, Science and Technology Innovation Action Plan (NO. 17511110300).

## Conflict of Interest

The authors declare that the research was conducted in the absence of any commercial or financial relationships that could be construed as a potential conflict of interest.

## Supplementary Material

Supplementary Figure 1 Network topology for soft-thresholding powers. The power numbers in the plots indicate soft thresholding value. The approximate scale-free topology can be selected by the power=4.

Supplementary Table 1 REGRNA analysis, Starbase analysis, miR-29a targets analysis and ILF3-AS1 co-expression information.

Supplementary Table 2 The clinical information of all GC patients.
