# Corrigendum: WGCNA Co-Expression Network Analysis Reveals ILF3-AS1 Functions as a CeRNA to Regulate PTBP1 Expression by Sponging miR-29a in Gastric Cancer

**DOI:** 10.3389/fgene.2020.585190

**Published:** 2020-10-07

**Authors:** Zhen-Hu Ren, Gao-Pan Shang, Kun Wu, Chuan-Yu Hu, Tong Ji

**Affiliations:** ^1^Department of Oral and Maxillofacial & Head and Neck Oncology, Shanghai Ninth People's Hospital, Shanghai Jiao Tong University School of Medicine, Shanghai, China; ^2^Department of Neonatology, Children's Hospital of Fudan University, Shanghai, China; ^3^Stomatology Center, Tongji Hospital, Tongji Medical College, Huazhong University of Science and Technology, Wuhan, China

**Keywords:** gastric cancer, WGCNA, co-expression network, miRNA, prognosis

In the original article, there was a mistake in the legend for Figure 6 as published. The terms “NSCLC” should be “GC” and “invasion” should be “migration”. The correct legend appears below:

**Figure 6:**
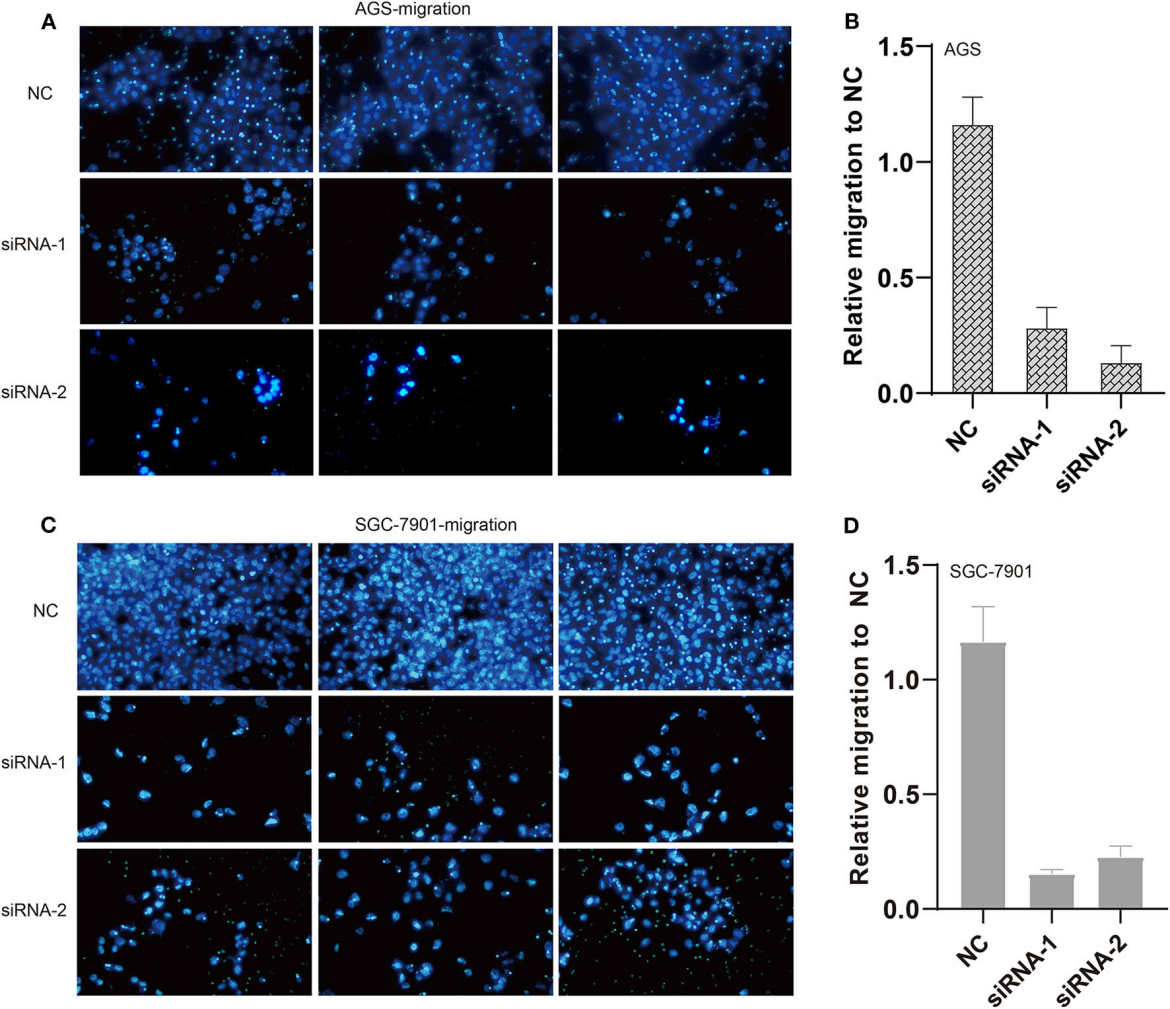
Knockdown of ILF3-AS1 induced cell migration in GC. **(A,B)** Knockdown of ILF3-AS1 induced cell migration in AGS cells. **(C,D)** Knockdown of ILF3-AS1 induced cell migration in SGC-7901 cells.

**FIGURE 6** | Knockdown of ILF3-AS1 induced cell migration in GC. **(A,B)** Knockdown of ILF3-AS1 induced cell migration in AGS cells. **(C,D)** Knockdown of ILF3-AS1 induced cell migration in SGC-7901 cells.

There was also a mistake in the legend for **Figure 7**. The term “NSCLC” should be “GC”. The correct legend appears below:

**FIGURE 7** | Knockdown of ILF3-AS1 induced cell invasion in GC. **(A,B)** Knockdown of ILF3-AS1 induced cell invasion in AGS cells. **(C,D)** Knockdown of ILF3-AS1 induced cell invasion in SGC-7901 cells.

Lastly, there was a mistake in Figure 6A (siRNA-2) as published. The correct figure is provided below.

The authors apologize for this error and state that this does not change the scientific conclusions of the article in any way. The original article has been updated.

