# Supplementary figures and images for: WGCNA Co-Expression Network Analysis Reveals ILF3-AS1 Functions as a CeRNA to Regulate PTBP1 Expression by Sponging miR-29a in Gastric Cancer
Source: Front Genet. 2020 Feb 14;11:39. doi: 10.3389/fgene.2020.00039 (PMC7033569; doi:10.3389/fgene.2020.00039)

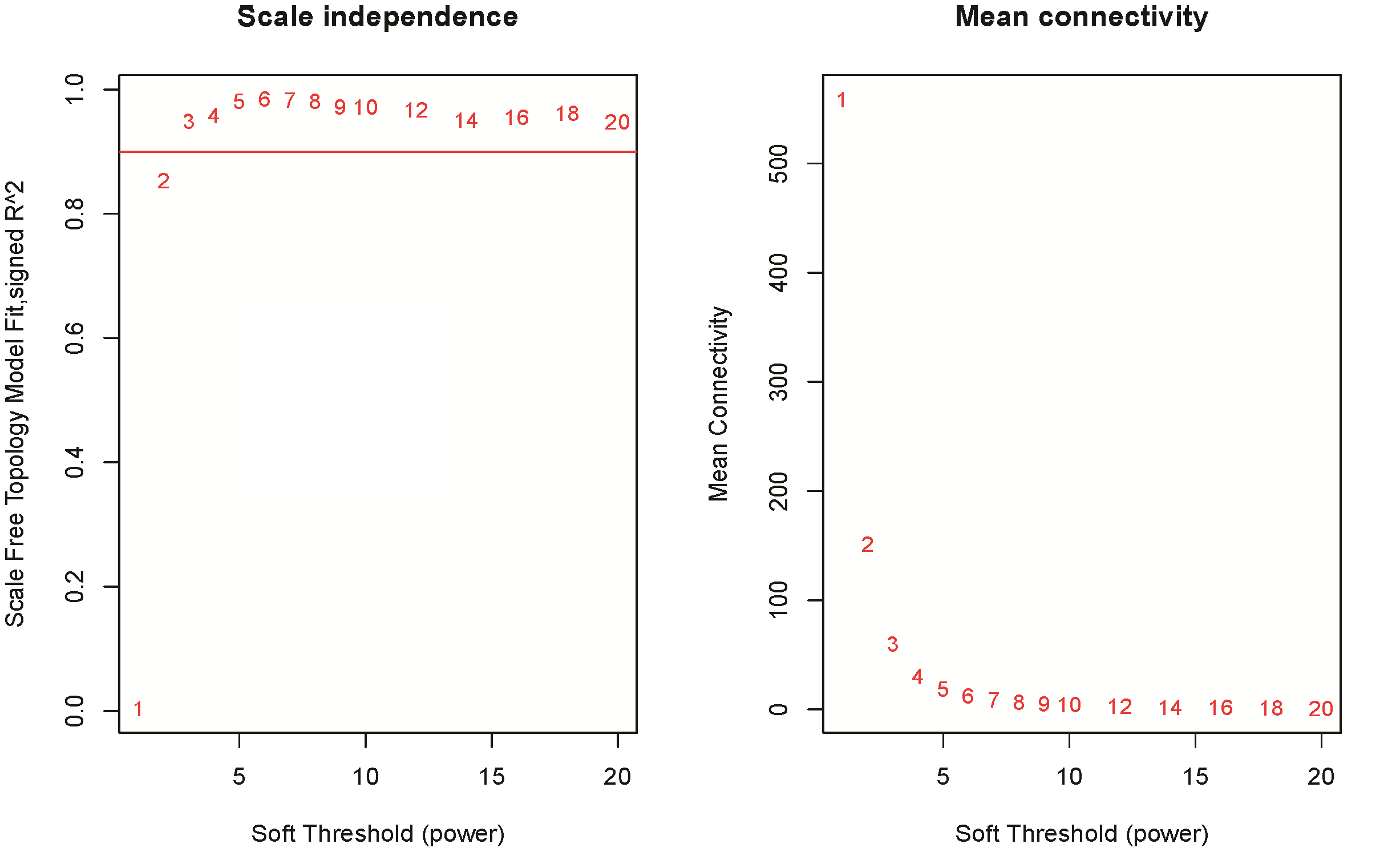

Supplement: Supplementary Figure 1 — Network topology for soft-thresholding powers. The power numbers in the plots indicate soft thresholding value. The approximate scale-free topology can be selected by the power=4. [file Image_1.tif]
